# Functional implications of glycans and their curation: insights from the workshop held at the 16th Annual International Biocuration Conference in Padua, Italy

**DOI:** 10.1093/database/baae073

**Published:** 2024-08-13

**Authors:** Karina Martinez, Jon Agirre, Yukie Akune, Kiyoko F Aoki-Kinoshita, Cecilia Arighi, Kristian B Axelsen, Evan Bolton, Emily Bordeleau, Nathan J Edwards, Elisa Fadda, Ten Feizi, Catherine Hayes, Callum M Ives, Hiren J Joshi, Khakurel Krishna Prasad, Sofia Kossida, Frederique Lisacek, Yan Liu, Thomas Lütteke, Junfeng Ma, Adnan Malik, Maria Martin, Akul Y Mehta, Sriram Neelamegham, Kalpana Panneerselvam, René Ranzinger, Sylvie Ricard-Blum, Gaoussou Sanou, Vijay Shanker, Paul D Thomas, Michael Tiemeyer, James Urban, Randi Vita, Jeet Vora, Yasunori Yamamoto, Raja Mazumder

**Affiliations:** Department of Biochemistry & Molecular Medicine, The George Washington University School of Medicine and Health Sciences, 2300 I St. NW, Washington, DC 20052, United States; York Structural Biology Laboratory, Department of Chemistry, University of York, Wentworth Way, York YO10 5DD, United Kingdom; The Glycosciences Laboratory, Imperial College London, Hammersmith Campus, Du Cane Road, London W12 0NN, United Kingdom; Glycan and Life Systems Integration Center (GaLSIC), Soka University, 1-236 Tangi-machi, Hachioji, Tokyo 192-8577, Japan; Department of Computer and Information Sciences, University of Delaware, 18 Amstel Ave, Newark, DE 19716, United States; Swiss-Prot Group, Swiss Institute of Bioinformatics (SIB), CMU, 1 rue Michel Servet, Geneva 4 1211, Switzerland; National Center for Biotechnology Information, National Library of Medicine, National Institutes of Health, 8600 Rockville Pike, Bethesda, MD 20894, United States; Michael Smith Laboratories, The University of British Columbia, 2185 East Mall, Vancouver, British Columbia V6T 1Z4, Canada; Department of Biochemistry and Molecular & Cellular Biology, Georgetown University, 2115 Wisconsin Ave NW, Washington, DC 20007, United States; Department of Chemistry and Hamilton Institute, Maynooth University, Kilcock Road, Maynooth, Co. Kildare W23 AH3Y, Ireland; The Glycosciences Laboratory, Imperial College London, Hammersmith Campus, Du Cane Road, London W12 0NN, United Kingdom; Proteome Informatics Group, Swiss Institute of Bioinformatics (SIB), route de Drize 7, Geneva CH-1227, Switzerland; Department of Chemistry and Hamilton Institute, Maynooth University, Kilcock Road, Maynooth, Co. Kildare W23 AH3Y, Ireland; Copenhagen Center for Glycomics, Department of Cellular and Molecular Medicine, Faculty of Health Sciences, University of Copenhagen, Blegdamsvej 3, Copenhagen DK-2200, Denmark; ELI Beamlines Facility, The Extreme Light Infrastructure ERIC, Za Radnicí 835, Dolní Břežany 25241, Czech Republic; IMGT, The International ImMunoGeneTics Information System, National Center for Scientific Research (CNRS), Institute of Human Genetics (IGH), University of Montpellier (UM), 141 rue de la Cardonille, Montpellier 34 090, France; Proteome Informatics Group, Swiss Institute of Bioinformatics (SIB), route de Drize 7, Geneva CH-1227, Switzerland; The Glycosciences Laboratory, Imperial College London, Hammersmith Campus, Du Cane Road, London W12 0NN, United Kingdom; Institute of Veterinary Physiology and Biochemistry, Justus-Liebig-University Gießen, Frankfurter Str. 100, Gießen 35392, Germany; Department of Oncology, Lombardi Comprehensive Cancer Center, Georgetown University Medical Center, 3900 Reservior Road NW, Washington, DC 20007, United States; European Molecular Biology Laboratory, European Bioinformatics Institute (EMBL-EBI), Wellcome Genome Campus, Hinxton, Cambridge CB10 1SD, United Kingdom; European Molecular Biology Laboratory, European Bioinformatics Institute (EMBL-EBI), Wellcome Genome Campus, Hinxton, Cambridge CB10 1SD, United Kingdom; Department of Surgery, Beth Israel Deaconess Medical Center, National Center for Functional Glycomics, Harvard Medical School, 330 Brookline Avenue, Boston, MA 02215, United States; Departments of Chemical & Biological Engineering, Biomedical Engineering and Medicine, University at Buffalo, State University of New York, 906 Furnas Hall, Buffalo, NY 14260, United States; European Molecular Biology Laboratory, European Bioinformatics Institute (EMBL-EBI), Wellcome Genome Campus, Hinxton, Cambridge CB10 1SD, United Kingdom; Complex Carbohydrate Research Center, University of Georgia, 315 Riverbend Rd, Athens, GA 30602, United States; Institute of Molecular and Supramolecular Chemistry and Biochemistry (ICBMS), UMR 5246, University Lyon 1, CNRS, 43 Boulevard du 11 novembre 1918, Villeurbanne cedex F-69622, France; IMGT, The International ImMunoGeneTics Information System, National Center for Scientific Research (CNRS), Institute of Human Genetics (IGH), University of Montpellier (UM), 141 rue de la Cardonille, Montpellier 34 090, France; Department of Computer and Information Sciences, University of Delaware, 18 Amstel Ave, Newark, DE 19716, United States; Department of Population and Public Health Sciences, University of Southern California, 2001 N Soto Street, Los Angeles, CA 90032, United States; Complex Carbohydrate Research Center, University of Georgia, 315 Riverbend Rd, Athens, GA 30602, United States; Department of Chemistry and Molecular Biology, University of Gothenburg, Medicinaregatan 7 B, Gothenburg 41390, Sweden; Immune Epitope Database and Analysis Project, La Jolla Institute for Allergy & Immunology, 9420 Athena Circle, La Jolla, CA 92037, United States; Department of Biochemistry & Molecular Medicine, The George Washington University School of Medicine and Health Sciences, 2300 I St. NW, Washington, DC 20052, United States; Database Center for Life Science, Joint Support-Center for Data Science Research, Research Organization of Information and Systems, 178-4-4 Wakashiba, Kashiwa, Chiba 277-0871, Japan; Department of Biochemistry & Molecular Medicine, The George Washington University School of Medicine and Health Sciences, 2300 I St. NW, Washington, DC 20052, United States

## Abstract

Dynamic changes in protein glycosylation impact human health and disease progression. However, current resources that capture disease and phenotype information focus primarily on the macromolecules within the central dogma of molecular biology (DNA, RNA, proteins). To gain a better understanding of organisms, there is a need to capture the functional impact of glycans and glycosylation on biological processes. A workshop titled “Functional impact of glycans and their curation” was held in conjunction with the 16th Annual International Biocuration Conference to discuss ongoing worldwide activities related to glycan function curation. This workshop brought together subject matter experts, tool developers, and biocurators from over 20 projects and bioinformatics resources. Participants discussed four key topics for each of their resources: (i) how they curate glycan function-related data from publications and other sources, (ii) what type of data they would like to acquire, (iii) what data they currently have, and (iv) what standards they use. Their answers contributed input that provided a comprehensive overview of state-of-the-art glycan function curation and annotations. This report summarizes the outcome of discussions, including potential solutions and areas where curators, data wranglers, and text mining experts can collaborate to address current gaps in glycan and glycosylation annotations, leveraging each other’s work to improve their respective resources and encourage impactful data sharing among resources.

**Database URL**: https://wiki.glygen.org/Glycan_Function_Workshop_2023

## Introduction

Biocuration has been foundational and an integral part of bioinformatics since Margaret O. Dayhoff demonstrated how collecting, analyzing, and annotating protein sequences can lead to a better understanding of the nature and function of the protein [1]. She and her co-worker’s effort also demonstrated the value of collection, standardization, and curation in an era that had already started producing significant amounts of data from experimental approaches to the study of life and function of biomolecules [2]. Defining the “function” of biomolecules, the building blocks of life, such as proteins, nucleic acids, lipids, and carbohydrates, has been elusive due to their contextual nature. Often biomolecules exist as conjugates or they interact with each other resulting in processes that are tightly coupled with the conditions they are exposed to. Therefore, it requires a collaborative effort from all spheres of biocuration to come together and discuss how glycan function is perceived within each knowledge domain. Such discussions can lead to common standards and definitions, which can accelerate our ability to leverage the different aspects of biocuration activities worldwide and can help us to better represent glycan function in databases and knowledgebases.

### Glycan function and how it relates to protein and gene function

The central dogma of molecular biology elegantly defined the relationship between gene, transcript, and protein as a highly regulated, but largely linear process that decodes template molecules into functional products. Many post-translational protein modifications lie outside of this dogma because they are not encoded by a template and usually reflect the convergence of various signaling pathways or other cellular responses to tissue microenvironments, disease, or developmental progression. The glycosylation process, defined as the synthesis, conjugation, and remodeling of glycans attached to other molecules (primarily proteins and lipids [3, 4], but recently also on RNA [5]), is a form of such nontemplate-driven biomolecule production. The glycans attached to proteins frequently modulate their folding, intrinsic stability, activity, and lifetime in circulation or at the cell surface [6]. They also influence viral- and microbial–host interactions, immune responses to pathogens, as well as pro- and anti-inflammatory status [7]. Detailed studies of individual glycans, their recognition by receptors, and their influence on carrier proteins can provide clues to their function. For each of these functions, the fine structure of the glycan itself and the specific amino acid residue to which the glycan is attached (the glycosylation site) combine to achieve observable biological responses. Expanding knowledge of glycosylation pathways, coupled with rapid advances in analytic technology over the past decade, have made it ever easier to describe the range of glycans expressed by specific cells or tissues and the diversity of glycans linked to specific glycosylation sites on identified proteins. These datasets have provided the raw material to develop knowledgebases and new opportunities to map glycosylation data to other types of data that together have vast potential to contribute to new understandings of biological functions.

### Challenges in curating glycan function

Many resources include glycan-related genes and proteins, such as biosynthetic and degradative enzymes, within their scope. However, function and disease annotations are usually associated with the gene or gene product rather than with glycosylation or glycan structural changes that might characterize cellular or tissue states. Despite the immense volume of glycoproteomic and glycomic data generated over the past decades, glycan function annotations in databases and knowledgebases have lagged behind by fundamental challenges in data communication, standards, and curation.

#### Data communication

Bench scientists are primarily trained to use their laboratory methods to answer specific questions. Their main objectives are to generate data and publish their findings based on accepted practices within their fields. However, these practices are designed to prioritize data presentation for publication and not to facilitate appropriate cataloging for effective curation. Often, information required and expected by curators is not immediately at hand or it takes significant effort for scientists to re-catalog data into a format accessible by curators. In addition, data tables in publications can be prone to errors associated with manual entry, and sometimes the author’s assertions are not verifiable based on the data presented in the manuscript.

#### Standards

Existing standard identifiers provided by various sources (e.g. GlyTouCan [8], ChEBI [9], PubChem [10]) represent glycans, but the adoption of these IDs by researchers has been slow. Currently, no journal requires the use of any of these standard glycan IDs. Some journals, including Glycobiology, recommend authors to follow the MIRAGE (Minimum Information Required for A Glycomics Experiment) guidelines [11] (https://www.beilstein-institut.de/en/projects/mirage/guidelines/). MIRAGE also recommends various glycan-related databases such as GlyTouCan, GlycoPOST, and UniCarb-DR as repositories to obtain accession numbers for glycans and analytical data (https://www.beilstein-institut.de/en/projects/mirage/recommendations/). Beyond standard glycan identifiers, several formats exist to describe glycan sequences, making it difficult for curators to map published sequence information to database IDs. In publications, human-readable International Union of Pure and Applied Chemistry (IUPAC) formats or the Symbol Nomenclature for Glycans (SNFG) graphics predominate [12], whereas databases store glycans using machine-readable formats such as GlycoCT [13] or WURCS [14, 15]. While converters have been developed (GlycanFormatConverter 2.7.0 https://glyconavi.org/Tools/tool/gfc.php, MolWURCS https://github.com/glycoinfo/Executable/tree/master/MolWURCS), they might not be compatible with all file formats and interconversion between formats used in publications to those applied by databases is often difficult. For instance, SNFG is quickly learned and easily read by humans, but difficult to parse programmatically because of its graphical nature. While IUPAC notation can be displayed as graphics or text, even the textual representations frequently fail to explicitly state information on absolute configuration, ring type, and anomericity. Thus, structural details have to be added by curators, who often need to infer the context to correctly assign these data. This need for additional interpretation also applies to SNFG representations, where anomers or linkage positions are not always explicitly specified but mentioned in the text.

#### Curation

Although funding bodies and journals have broadly encouraged scientists to deposit all types of data into public repositories, curation always requires significant effort and care. Curating glycan data takes even more effort due to the variable nature of the primary data and the intrinsic complexity of glycan structures. Coordination among resources to curate biomolecules within the context of glycan function has been lacking, especially in comparison to the coordination between resources specialized for curating gene and protein functions. This disparity may not be broadly appreciated but needs to be addressed in order to help build robust connections between glycoscience data and more mature bioinformatic domains. Resources and funds are not readily available to support such efforts, especially to curate legacy data, resulting in an environment where curation (or even presentation of data in a curatable manner) is not prioritized. Ideally, publication should not be the only incentive to curate and deposit data, as there is a considerable amount of data that never gets published or deposited in an accessible data resource (e.g. data orphaned from abandoned projects or data supporting negative hypotheses).

To address these challenges and improve glycan functional annotations and standards, a whole-day workshop on the functional impact of glycans and their curation was held in conjunction with the 16th Annual International Biocuration Conference in Padua, Italy, on 23 April 2023.

## Workshop summary

**Figure 1.**
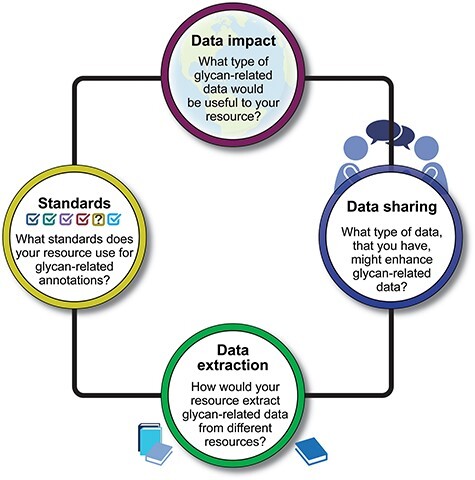
A visual summary of four key questions posed during the workshop. The responses to these questions provide insights into the diverse approaches and strategies employed by the workshop participants in addressing crucial aspects of glycan function annotations.

Bioinformaticians, curators, and glycobiologists from over 20 projects and bioinformatics resources that contain glycan-related data participated in presentations and discussions (Table 1 and Supplementary File 1). Participants were invited to share their perspectives, curation workflows, bottlenecks, and needs for extracting and disseminating knowledge about glycans and their function. With a focus on biocuration, data sharing, and data harmonization, the workshop was organized around four questions (Fig. 1), each presented here along with a summary of the discussion, findings, and proposals for future improvements.

**Table 1.** Resources and projects represented at the workshop

Resource/project	Resource type	URL	Latest publication PMID/URL	Participant/s
BioCreative	Standard	https://biocreative.bioinformatics.udel.edu/	36197453	Cecilia Arighi
ChEBI	Knowledgebase, Standard	https://www.ebi.ac.uk/chebi/	26467479	Adnan Malik
Glyco.me GlycodomainViewer	Knowledgebase	https://glyco.me/ https://glyco.me/glycodomain/	29267884	Hiren Joshi
Glyco@Expasy	Tool	https://glycoproteome.expasy.org/glycomics-expasy/	30097532	Frederique Lisacek
GlycoEnzOnto	Standard	https://github.com/neel-lab/GlycoEnzOnto	36282863	Ted Groth, Sriram Neelamegham (remote)
Glycomotif GlyGen Sandbox	Knowledgebase	http://glycomotif.glyomics.org/ http://sandbox.glyomics.org/	N/A	Nathan Edwards
GlyConnect	Knowledgebase	https://glyconnect.expasy.org/	30574787	Frederique Lisacek
Glycosciences.de MonosaccharideDB	Knowledgebase	www.glycosciences.de www.monosaccharidedb.org	30357361	Thomas Lütteke (remote)
GlycoShape	Knowledgebase	https://glycoshape.org/	https://doi.org/10.1101/2023.12.11.571101	Callum Ives, Elisa Fadda (remote)
GlyCosmos	Knowledgebase	https://glycosmos.org	32572234	Kiyoko F. Aoki-Kinoshita, Yasunori Yamamoto
Glycowork	Knowledgebase	https://github.com/BojarLab/glycowork/	34192308	James Urban
GlyGen	Knowledgebase	https://www.glygen.org	31616925	Karina Martinez, René Ranzinger, Vijay Shanker, Nathan Edwards, Michael Tiemeyer, Raja Mazumder, Jeet Vora (remote)
GNOme	Standard	http://gnome.glyomics.org/	https://ceur-ws.org/Vol-3073/paper11.pdf	Nathan Edwards
GO	Standard	https://geneontology.org/	36866529	Paul Thomas
ICL Glycan Microarrays	Knowledgebase	https://www.imperial.ac.uk/glycosciences/ https://glycosciences.med.ic.ac.uk/glycanLibraryIndex.html	N/A	Ten Feizi, Yan Liu, Yukie Akune
IMGT	Knowledgebase	https://www.imgt.org/	34875068	Taciana Manso (remote), Sofia Kossida (remote)
Immune Epitope Database and Analysis Project (IEDB)	Knowledgebase	https://www.iedb.org/	30357391	Randi Vita
IntAct	Knowledgebase	https://www.ebi.ac.uk/intact	34761267	Kalpana Panneerselvam
iPTMnet	Knowledgebase	https://research.bioinformatics.udel.edu/iptmnet/	29145615	Cecilia Arighi
MatrixDB	Knowledgebase	http://matrixdb.univ-lyon1.fr/	30371822	Sylvie Ricard-Blum (post-workshop)
NCFG—Glybrary	Tool	https://www.glybrary.com	N/A	Akul Mehta (remote)
O-GlcNAcAtlas OGT-PIN	Knowledgebase	https://oglcnac.org/atlas/ https://oglcnac.org/ogt-pin	33442735	Junfeng Ma (remote)
PRIDE	Archive	https://www.ebi.ac.uk/pride/	34723319	Deepti Jaiswal Kundu
Privateer	Tool, Knowledgebase	https://github.com/glycojones/privateer, https://privateer.york.ac.uk/	26581513, 38711584, 38265073	Jon Agirre (remote)
PubChem	Archive, Knowledgebase	https://pubchem.ncbi.nlm.nih.gov	36305812	Evan Bolton (remote)
Rhea	Knowledgebase	https://www.rhea-db.org/	34755880	Kristian Axelsen, Alan Bridge (remote)
SNFG	Standard	https://www.ncbi.nlm.nih.gov/glycans/snfg.html	31184695	Sriram Neelamegham (remote)
TogoID	Tool	https://togoid.dbcls.jp/	35801937	Yasunori Yamamoto
UniLectin	Knowledgebase	https://unilectin.unige.ch/	33174598	Frederique Lisacek, Anne Imberty
UniProtKB	Knowledgebase	https://www.uniprot.org	36408920	Maria Martin, Cecilia Arighi, Kristian Axelsen, Alan Bridge (remote)

N/A indicates data are not available.

### Q1. How would your resource extract glycan-related data from literature or other sources?

Advances in experimental techniques and growth in the field of glycobiology have led to an influx of scientific publications and data related to glycans and glycosylation over the past decade. As described in the previous section, several challenges exist in extracting and curating this data. To gain consensus and better understand how resources handle these challenges, the first session addressed how resources extract glycan-related data from the literature and other sources. Participants focused on identifying strategies for data extraction, needs and bottlenecks, curation workflow, common solutions, and areas of improvement.

#### Curation

In most cases, information extraction is performed by expert human curators who read the publication and extract relevant data. Literature and text mining tools are often used to assist in identifying relevant publications, such as LitSuggest [16], PubTator [17], and those created by the resources themselves.

Rhea [18] and collaborators at US National Center for Biotechnology Information (NCBI) are developing an expert curated corpus to train and benchmark methods for extracting enzyme functions from text. This corpus was curated using the TeamTat framework [19] and using procedures developed for the BioRED corpus; it has shown promising results when used to fine-tune models such as BioREx [20]. The Imperial College London (ICL) Glycosciences Lab is creating a novel curated glyco-interactome resource that aims to integrate knowledge on glycan-mediated biomolecular interactions from glycan microarray datasets. They are collaborating with Joram Posma at ICL to establish a text mining approach based on Auto-CORPus [21], a tool originally developed for metabolites. GlyGen [22], in collaboration with Vijay Shankar at the University of Delaware, developed text mining tools to create a dictionary of glycan structure terms [23]. Plans include using this dictionary to extract information related to glycan function from the literature. Automated text mining results from the mining of abstracts are available in the glycosylation section of the GlyGen protein pages. IntAct [24] uses text mining-based screening in PubMed and Europe PubMed Central (EPMC) [25]. They are working with EPMC literature services to develop a screening portal that aims to use a combinatorial text mining and machine learning-based strategy. Glyco@Expasy [26] uses a combination of text mining tools and manual opportunistic selection to identify sources. Depending on the format of the supplementary material in publications, data extraction ranges from manual to semiautomatic. UniProtKB [27] captures data from the literature by expert annotation. Proteins are annotated when a relevant publication is identified, or if experimental information about glycosylation sites is present. N-glycosylation sites are mainly curated based on prediction tools, but the annotations are not propagated to close homologs. O-glycosylation sites for which there is information about the function may be propagated.

Many curation workflows also include the integration of data from other resources. IntAct partners with MatrixDB [28], which is actively involved in the manual curation of glycosaminoglycan (GAG)–protein interactions. iPTMnet [29] captures sites of protein glycosylation from external resources including UniProtKB, GlyGen, SIGNOR [30], and others. VirtualGlycome (https://VirtualGlycome.org) aims to manually collect data from sources including textbooks, handbooks, research papers, and published datasets from UniProtKB, GlyGen, NCBI, KEGG [31], and others. Data are processed in a field/topic-specific manner to obtain results that are curated by VirtualGlycome. GlyGen retrieves and harmonizes data related to glycoscience from both individual collaborators and public databases, such as UniProtKB, GlyTouCan, GlyConnect [32], O-GlcNAcAtlas [33] and the O-GlcNAc Database [34]. At ChEBI, data are submitted to the resource by the user community or other resources such as Rhea, GlyGen, MetaboLights [35], and others.

#### Validation and verification

Participants described various ways to validate and verify the quality of annotations. In some cases, this involves a two-step curation process in which annotations are checked by a second biocurator. For others, this mechanism is too costly or creates a bottleneck, and systematic automated checks based on manually created rules are put in place to detect common errors. Some resources try to correct errors when they encounter them, such as inconsistencies in describing alpha versus beta linkages. Others rely on assertions made by the original author and follow consistent rule sets when annotating publications.

The two-step protocol described above is employed by IntAct, MatrixDB, and Rhea, where reactions are created by one curator and verified by a second. SugarBase [36] manually annotates their entries with species, tissue, and disease associations, including glycan structure dysregulation in disease. Programmatic verification is subsequently used to check entries before merging with the SugarBase database. Glycan microarray data curation at ICL will be facilitated by machine learning-based semiautomatic extraction of experimental data that are compliant with the MIRAGE glycan microarray guidelines and followed by manual curation by specialists. GlyGen uses a combination of manual and automated checks that include verifying that glycosylation sites match the correct amino acid position in the UniProtKB canonical sequence and mapping nonstandard identifiers to their primary protein or glycan keys. When data are submitted to ChEBI, a stable and unique identifier is assigned to each submission, and the submitted metadata is subsequently checked and manually annotated by the ChEBI team.

#### Q1 conclusions

Overall, extraction of glycan-related data from the literature is largely a manual process and requires expert curators with domain knowledge in glycobiology. While text mining tools are used to identify papers of interest, information extraction is still performed by a human curator in most resources. Current tools are limited in scope and focus on a specific type of data, such as array data or glycosylation site annotation. Once data extraction and standardization are complete, programmatic tools are often used to assist curators in verifying the information. However, these verification tools or pipelines are custom-made by individual groups and there is no generalized approach allowing reuse of these tools for more than one curation initiative. It appears that close collaboration among the participating groups could lead to joint curation of publications which in turn can help in text mining efforts. Better coordination between curation initiatives would benefit the entire ecosystem of resources in this space by creating tools and workflows that can be easily adapted to multiple purposes.

### Q2. What type of glycan-related data would be useful to your resource, that you do not currently have access to?

Glycan data exist in various resources but may not be accessible due to inconsistencies in standards and interpretation. In addition to the resources involved in this effort, glycan-related data exist in various omics data resources (e.g. metabolomics and lipidomics) which need to be parsed and annotated. In this session, participants discussed the types of glycan-related data they would like to have access to and would enhance their resources. Resources compared data of interest and challenges to find common ground and opportunities to improve data sharing and accessibility.

#### Knowledge representation

The Immune Epitope Database (IEDB) [37] is interested in the connection between structures with epitopes, organisms by which the epitopes are produced, structures and organisms that contain the epitopes, and similarities between epitope structures across all species. They expressed that immunologists would benefit from systematic classifications of glycan-related data based on biology. Emphasis was placed on the need for effective linking to glycan resources and the availability of glycan-related annotations and relationships between structures and organisms in an Open Biological and Biomedical Ontology (OBO) [38] compatible format to allow reasoning and interoperability. GlycoMotif (https://glycomotif.glyomics.org) and GlyGen Sandbox (https://sandbox.glyomics.org) would benefit from glycan-related functional annotation terms from data sources outside the glycobiology domain. Challenges include indirect, non-glyco function for glyco-enzymes (e.g. glycan does not modulate the protein function) and lack of glyco-function terms, such as Gene Ontology (GO) Molecular Function terms for proteins. GlyGen suggested the development of a glycan function ontology describing the glycan field on the model of GO and how that can be useful to better explore and contextualize glycomic datasets. Glycan biology is currently captured in the GO [39, 40] as a function of proteins and ncRNAs. GO collaborates with experts in specific areas of biology to revise the ontology and add protein annotations, and would require a community effort to drive utility in glycobiology. To initiate a discussion in this area, they suggested that the community consider how they would like to model glycan functions. For example, are they conceptualized as molecular machines like proteins, mediators of specific molecular recognition by proteins, and/or post-translational modifications that modulate protein functions?

#### Interactions

A multitude of processes in health and most diseases critically involve glycans, directly or indirectly. With the increasing amounts of glycan microarray data in scientific publications, there is a great need to organize published and curated data; this together with other forms of glycan interaction data will serve as a much-needed knowledgebase for the broad scientific community. The ICL Glycosciences Lab emphasized that it would be beneficial to integrate the curated glycan interaction data with the expression of the glycan ligands and their carrier molecules, ideally within the microenvironment where the biorecognition event occurs. This integration would lead to a deeper understanding of glycan-mediated interactions and elucidation of biological pathways in relation to other disciplines. IntAct described the integration in MatrixDB of curated GAG interaction datasets collected by several techniques, including affinity proteomics to identify GAG-binding proteins in various cell types, subcellular compartments, extracellular matrices, and biological fluids in health and disease, to generate context-dependent GAG interactomes, and to contextualize the global GAG interactome 2.0 [41]. The integration of the composition, and ideally of the sequences, of GAGs interacting with proteins would be useful to decipher the molecular recognition mechanisms of GAGs by proteins, and to link them to GAG and protein functions.

#### Structural representation

Visual representations of glycans and reactions, and tools to map cheminformatic representations to graphical representations (e.g. SMILES [42] to SNFG), would be of interest to several resources. Rhea works closely with ChEBI and requires the structures of substrates and products to construct reactions; however, it is not always easy to find the precise structures in the papers. ChEBI does not currently display glycan structures using the SNFG system; however, the process and tools to curate such data into ChEBI would be welcomed. GlycoEnzOnto [43] would like to more tightly integrate glycan structural data with their ontology to semantically link glycoenzyme function to the glycan structures they produce. Integration of GNOme, GlycoEpitope, GlycoTree, and GlyGen’s glycan list could make this connection.

#### New data

Most groups expressed interest in collecting new data and annotations to enrich their collections, as well as more contextual information on the glycan sources, such as species, tissues, cell lines, instrumentation, disease, age, and sex. Attending glycobiologists commented that more data on site occupancy, glycan structure, and abundances (even if just relative) would be very useful. UniProtKB is focused on sites of protein glycosylation and capturing types of attachment using an internally controlled vocabulary, especially those with functional impact. As part of the ongoing standardization of all small molecule data in UniProtKB, the PTM vocabulary list (https://ftp.uniprot.org/pub/databases/uniprot/current_release/knowledgebase/complete/docs/ptmlist.txt) is being progressively mapped to ChEBI. iPTMnet would like to include text mining results with protein, site, and glycan information. Other resources such as PRIDE [44] are interested in well-annotated glycoproteomic data, and Privateer [45] would benefit from having access to in-depth glycomics data, particularly for uncharacterized systems (e.g. extremophiles), as glycomics results are being used to validate or support 3D structures of glycans for scientists who may not necessarily be experts in glycobiology.

X-ray crystallography is the preferred tool to determine the high-resolution structures of glycan and glycan–protein complex structures [46, 47]. Although these systems are understood as not being easy targets, recent progress in cryo-EM and cryo-electron diffraction has significantly increased the number of glycan–protein complex structures solved [48, 49]. The evolution of these methods carries the potential to generate a significant amount of structural data of the glycan and glycan–protein complexes. This advance would enrich the experimentally obtained 3D structures of glycan/glycoproteins instead of solely relying on glycan structures from molecular dynamics simulations.

### Q2 conclusion

The resources agreed on the importance of knowledge exchange and the need for curation guidelines when considering glycan functions. Machine-readable annotations are lacking for glycan-related data and terms similar to those in GO. Such terms would facilitate linking across resources and connect knowledge of glycan structures, glyco-enzymes, organisms, and other types of information. Assignment of glycan structures to wet lab experimental data (e.g. MS spectra, lectin arrays, etc.) is variable across the scientific community. This inconsistency is partially due to the lack of reporting standards, but largely due to idiosyncrasies of data interpretation. Currently, “reliable” glycan-recognition data that can be curated into databases is somewhat limited and its interpretation can vary depending on the details of the wet lab experiment and interpretation of the data and the publication. Overlapping interests and opportunities to leverage existing frameworks and ontologies warrant further discussion and collaboration.

### Q3. What type of data, that you have, might enhance glycan-related data?

The primary aim of a bioinformatic resource is to provide a service or knowledge that will be beneficial to the research community. As the importance of glycans gains visibility outside the glycobiology community, the type and format of the data must be tailored for accessibility to both glycobiologists and non-glycobiologists alike. In the third session, participants focused on opportunities to share data and enhance their glycan-related data by discussing what type of data each resource has that is unique and might be useful to other resources. In addition to what is described below, details of what individual resources have to offer are available in Supplementary Table S1.

GlyGen provides text-mining data from manuscripts on experimentally determined glycosylation sites and has collected additional curated data through manual curation and collaboration with various other groups. Plans include adding ISOGlyP-predicted O-glycosylation sites [50]. O-GlcNAcAtlas [33] contains O-GlcNAc sites, peptides, and proteins. UniProtKB annotates N-linked glycosylation, O-linked glycosylation, C-linked glycosylation, S-linked glycosylation, and glycation using an internal controlled vocabulary. The identity of the reducing monosaccharide is indicated in the feature lines, while the monosaccharide composition is indicated in the PTM comments. The PTM keyword “Glycoprotein” is added to the protein entry when these annotations are present. In terms of leveraging different resources to gain knowledge, examples such as GlycoEnzOnto mappings to GO and UniProtKB were presented. Such mappings can contextualize glycoenzyme knowledge and IUPAC-based reaction rules describing glycoenzyme functions, which could be used to construct glycosylation reaction networks in the future.

Several tools and resources were mentioned that biocurators might find useful. Examples include publication annotations from PubAnnotation [51] that provide links to glycan structure images and GlyTouCan IDs from PubMed abstracts. The tools GlycoSim [52] and GlycoMaple [53] allow users to predict and visualize glycan biosynthesis pathways. MicroGlycoDB [54] is a new database containing glycogenes, reactions, pathways, and glycans in microbial species. ChEBI contains a chemical ontology, where glycans are classified based on chemical structure and roles. The resource is widely used and provides an infrastructure for connecting glycans to ChEBI ontology. PubChem contains a wealth of content that helps to define the boundaries of “what is a glycan” from a structural perspective. Glycowork [55] has a large number of tissue, disease, and species associations with glycans. Many come from older papers and other data sources, such as the Consortium for Functional Glycomics (CFG). Associations of this kind can be particularly useful to study glycan biosynthesis during evolution, tissue-specific expression, and to identify disease-specific markers.

In terms of interaction data, MatrixDB has built an automated pipeline to standardize the format of GAG sequences interacting with proteins manually curated from the literature. The pipeline then translates the sequences into machine-readable GlycoCT or SNFG images and converts them into a format processed by a builder. The builder generates 3D structures of GAGs based on a repertoire of conformations experimentally validated by data extracted from crystallized GAG–protein complexes [56]. In addition to the structural data listed above, MatrixDB provides glycosaminoglycan interaction data collected by manual curation of the literature following the curation rules of the International Molecular Exchange consortium [57, 58]. The ICL Glycosciences Lab proposed CarbArrayKB as a new resource centered on the curated glycan microarray database, which will serve as a knowledgebase for the broad scientific community, focusing on glycan-mediated interactions from high-quality publications conforming to MIRAGE guidelines. They are in discussion with EMBL-EBI with the hope of collaborating to integrate the glycan interaction data as an extension of IntAct (molecular interactions) and Reactome (biomolecular pathways). IMGT-KG is the pioneer of the immunogenetics knowledge graph [59]. It integrates and establishes connectivity with other standards, including Protein Data Bank (PDB) [60], IEDB, PubMed, and most recently, the GlyConnect platform. In IMGT-KG, a crystal structure is linked to a glycan through the relation “hasGlyConnectLink” and includes direct links to IMGT and PDB resources. The GlycoShape [61] platform provides a comprehensive open-access database of glycan structures from extensive molecular dynamics simulations. This platform provides a wealth of structural information that can be integrated and coordinated with other glycan-related resources. GlycoShape also provides users with many helpful structural tools, such as Re-Glyco, a bespoke algorithm in GlycoShape designed to rapidly restore the natural glycosylation to protein 3D structures and to predict N-glycosylation occupancy where unknown. Privateer validates data of 3D structures, torsional analysis, and cross-linking with some glycomics databases. One area for the future direction that was discussed is to annotate structures with matched entries across GlySpace Alliance resources [62].

#### Q3 conclusion

All attendees expressed interest in getting feedback on what data, tools, resources, and information will be useful for glycan function curation and allow for easier data sharing with other resources. It was agreed that closer collaboration with participating resources would increase data accessibility and create connections between different fields of research. Communication between researchers and the resources that desire access to their data remains a bottleneck. Creation of a clearing house for questions and recommendations regarding the deposition and curation of glycan-related data would be beneficial. Proper evidence attribution was emphasized as an essential feature for enhancing confidence in the usefulness of a resource when accessed by investigators less familiar with glycoscience data.

### Q4. What standards does your resource use for glycan-related annotations?

Standards improve interoperability and interconnectivity between resources and across domains. While some standards such as gene symbols, protein accession numbers, and glycan symbol nomenclature have converged to a few types, others such as glycan IDs and glycan structure text representation are still in flux. Resources use varying identifiers for glycans and their annotations depending on their biomolecules of interest and intended audience. This session focused on standards used by each resource and the challenges in identifying an ideal standard. A summary of standards currently used by the participating resources is presented in Table 2.

**Table 2.** Standards relevant to the curation of glycans that are currently in use by different projects and resources

	Primary standards and annotation target								
Resource/Project	Glycan ID	Glycan image	Glycan text representation	Protein ID/AC	Tissue	Disease	Cell lines	Taxonomy	Annotation target
ChEBI	ChEBI, PubChem CID, GlyTouCan, KEGG GLYCAN	MOL	IUPAC, WURCS	UniProtKB, PDB	Uberon, BRENDA	N/A	N/A	NCBI	Small molecules
Glyco.me	N/A	SNFG	N/A	UniProtKB	Uberon, BRENDA	N/A	Cellosaurus	NCBI	Glycosites and glycoproteins
Glyco@Expasy	GlyTouCan	SNFG	GlySTReeM, GlycoCT	UniProtKB	Uberon, BRENDA	DO	Cellosaurus, CLO	NCBI	Glycoproteins, glycan-binding proteins, glycans
GlycoEnzOnto	IUPAC-condensed	N/A	N/A	NCBI Accession, UniProtKB, EC number	N/A	N/A	N/A	NCBI	Glycosylation pathways
GlycoMotif	GlyTouCan	SNFG	WURCS, GlycoCT	UniProtKB, Mouse Genome Informatics (MGI), Gene name	N/A	MP	N/A	N/A	Glycans
Glycosciences.de	LINUCS ID	SNFG	LINUCS	PDB	N/A	N/A	N/A	NCBI	Glycans, proteins (PDB entries)
GlycoShape	GlyTouCan	SNFG	IUPAC-condensed, GLYCAM, WURCS	UniProtKB	N/A	N/A	N/A	N/A	Glycans
GlyCosmos	GlyTouCan	SNFG	WURCS	UniProtKB	Uberon	DO	CLO	NCBI	Proteins, glycans, glycoconjugates, glycogenes, lectins, glycolipids
Glycowork	GlyTouCan	SNFG	IUPAC-Condensed	N/A	Uberon	DO	CL	NCBI	Glycans
GlyGen	GlyTouCan	SNFG	GNOme	UniProtKB	Uberon	DO	Cellosaurus	NCBI	Proteins, glycans, glycosylation sites
GlyGen Sandbox	GlyTouCan	SNFG	GlycoCT	UniProtKB, Gene name	N/A	N/A	N/A	N/A	Glycans, glycosylation enzymes
GNOme	GlyTouCan	SNFG	N/A	N/A	N/A	N/A	N/A	N/A	Glycans
GO/PANTHER	N/A	N/A	N/A	UniProtKB	Uberon, Plant Ontology (PO), Fungal Anatomy Ontology (FAO)	N/A	CL	NCBI	Gene products (proteins, ncRNAs)
IEDB	ChEBI	N/A	N/A	UniProtKB	Uberon	DO	CLO	NCBI	Peptides, small molecules (<5000 Da)
IMGT	GlyConnect	N/A	N/A	PDB	N/A	Mondo	N/A	NCBI	Protein structural data
IntAct	ChEBI	N/A	N/A	UniProtKB	Uberon, BRENDA	Mondo	CLO Cellosaurus, EFO	NCBI	Mostly proteins, DNA, RNA, small molecules, and glycans
iPTMnet	N/A	N/A	N/A	UniProtKB	N/A	DO	N/A	NCBI	Proteins
MatrixDB	ChEBI, cross-reference to GlyTouCan when available	SNFG	GlycoCT	UniProtKB	Uberon, BRENDA	Mondo	CLO Cellosaurus, EFO	NCBI	Proteins and glycosaminoglycans
MIRAGE Glycan array guidelines	GlyTouCan (recommended)	SNFG (recommended)	ICL 2D Text, GlycoCT, (recommended)	Public database IDs (if available)	N/A	N/A	N/A	N/A	Glycan microarrays, glycans, glycan binding samples (including proteins and microorganisms)
NCFG—Glybrary	GlyTouCan, CFG Linear Nomenclature	SNFG	CFG Linear Nomenclature	UniProtKB	UniProtKB controlled vocabulary	Mondo	Cellosaurus	NCBI	Glycan microarrays and glycomics
oglcnac.org	O-GlcNAcAtlaS, OGT-PIN	N/A	N/A	UniProtKB	N/A	N/A	N/A	NCBI	O-GlcNAcylated sites/peptides/proteins
Privateer	GlyTouCan, GlyConnect	SNFG	N/A	N/A	N/A	N/A	N/A	N/A	Proteins, glycans
PubChem	CID, ChEBI, GlyTouCan	MOL, SNFG	IUPAC-condensed, LINUCS, WURCS, IUPAC	EC Number, NCBI Accession, RefSeq Accession, UniProtKB, PRO	Uberon	Medical Subject Headings (MeSH), International Classification of Diseases (ICD-11)	Cellosaurus, ChEMBL, Library of Integrated Network-based Cellular Signatures (LINCS), EFO, CLO, CL, BRENDA	NCBI, Integrated Taxonomic Information System (ITIS), Catalogue of Life (COL)	Small molecules, nucleotides, carbohydrates, lipids, peptides, and chemically-modified macromolecules
Rhea	ChEBI	MOL	IUPAC-extended	UniProtKB	N/A	N/A	N/A	N/A	Biochemical and transport reactions
SNFG	IUPAC	SNFG	N/A	N/A	N/A	N/A	N/A	N/A	Monosaccharides, glycans, glycoconjugates
UniProtKB	N/A	N/A	N/A	UniProtKB	Controlled vocabulary	Controlled vocabulary	N/A	NCBI	Proteins

Note: Acronyms not spelled out in this table are available in the manuscript text. N/A indicates data are not available.

#### Gene, protein, and taxonomy

Gene, protein, and taxonomic information associated with glycan data, such as genes involved in glycan biosynthesis, glycan-binding proteins, and species annotations, can be found across resources. There is broad consensus in using HUGO Gene Nomenclature Committee [63] for gene symbols for human genes, UniProtKB or RefSeq [64] for protein accession numbers, and PDB for 3D structures. NCBI taxonomy ID [65] is used across all participating resources as the primary taxonomy identifier.

#### Glycan images, identifiers, and descriptions

The SNFG was developed as a community effort to standardize graphical representations of glycan structures and has achieved wide acceptance in the glycobiology community. Almost all glycoinformatics resources present at the workshop, including PubChem, support the SNFG format. MOL format is used to represent glycan structures in chemical-centric resources such as ChEBI, Rhea, and PubChem. Some resources such as GlyConnect support Oxford notation [66] as well.

The majority of resources use either GlyTouCan or ChEBI identifiers for glycans. All GlySpace Alliance members use GlyTouCan identifiers and, whenever available, map to PubChem and ChEBI. GlyGen also assigns unique identifiers to their list of motifs and a dictionary of glycan structure terms [23], where the biocuration community can add new terms. ChEBI identifiers and annotations are used by Rhea, IntAct, IEDB, and others. ChEBI is aligned with the IUPAC Blue Book, IUPAC Gold Book, and NC-IUBMB Enzyme Commission numbers and uses standard practices for assigning chemical names, special characters, chemical structures, cross-reference links, ontologies, enzymes, and species. Reactions in Rhea, including glycans, are formally described using ChEBI vocabulary. However, glycan representation in Rhea is limited and work is ongoing with GlyGen and ChEBI to add glycans with publication references to ChEBI. IntAct and MatrixDB use ChEBI identifiers for GAG and oligosaccharides in agreement with the IMEx/IntAct curation rules. GAG–protein interactions are curated using Proteomics Standards Initiative Molecular Interaction and MI Ontology Lookup Service (https://www.ebi.ac.uk/ols4/ontologies/mi) controlled vocabularies. In general, many identifiers are used in biomedical databases, and ID conversion is needed to ensure interoperability. Tools such as TogoID [67] provide ID mapping between resources (e.g. PubChem and GlyTouCan) and can be accessed through the Web interface and Application Programming Interface.

Glycan sequences can be represented by glycan-specific notations such as WURCS and GlycoCT or by traditional chemical notations such as IUPAC, SMILES, InChI [68], and others. GlySpace Alliance members support WURCS and GlycoCT encoding while PubChem and ChEBI use InChI, SMILES, and IUPAC as the primary structural descriptors. Sequence converters allow resources to display both chemical and glycan-specific notations. For example, PubChem compounds include WURCS and LINUCS [69] sequences for biologics consisting of saccharides. Other participating resources use combinations of the aforementioned notations, except Glycowork, where a slightly modified version of IUPAC-condensed is used for glycan sequences, and MonosaccharideDB [70], which uses residue names (core monosaccharides + substituents) in various notations.

#### Ontology

Ontologies are used to describe relationships between terms or concepts in a way that is both human and machine-readable, enabling the organization of knowledge and interoperability between resources. There is consensus in the use of ontologies to describe protein biology, disease, cell, tissue, and anatomy, while glycan-centric ontologies have yet to proliferate into the bioinformatics space. The GO project creates terms that are the standard for describing the biological function, cellular component, and biological process of gene products, including glycosylated proteins. Several of the resources use GO terms to annotate protein entries including UniProtKB, Rhea, GlyConnect, and GlyGen. GlyGen also uses Protein Ontology (PRO) [71] annotations to describe proteoforms and glycoforms GlycoEnzOnto describes the organization of enzymes participating in cellular glycosylation. It is currently focused on ∼400 human glycosylating enzymes and related components. GlycoEnzOnto is based on the framework developed by GO and can be used for overrepresentation and pathway analysis.

A few glycan structure-specific ontologies and standards are currently in use. ChEBI has a manually curated ontology that is subdivided into three separate subontologies: Molecular structure, in which glycans are classified according to composition and structure; Role, in which glycans are classified based on their role within either a chemical, biological, and/or application context; and Subatomic Particle, which classifies particles smaller than atoms. ChEBI places carbohydrate structures into a hierarchy by making statements such as “β-D-glucose *is_a* D-glucose, which *is_a* D-aldohexose, which *is_a* monosaccharide, which *is_a* carbohydrate.” GNOme is an OBO foundry ontology for GlyTouCan identifiers covering the complete glycan space, including defined and undefined aspects. GlyGen uses the GNOme ontology for subsumption exploration and propagating annotations (including species and glycan classifications) from more characterized structures to less characterized structures. The PRO ontology uses the GNOme ontology to describe the glycosylation of protein isoforms. While there still exists variability among resources in glycan motif nomenclature, the GlycoMotif glycan determinant and motif resource unifies a variety of glycan motif lists in one place and provides precomputed alignments of all motifs with GlyTouCan accessions. Additional motifs are used to help classify glycan structures into types and subtypes. Recently, the enzyme annotations on glycan structures from the GlyGen Sandbox have been combined with motif alignments to structures to associate glycoenzymes with motif residues. Through this mechanism, mouse knockout results from the International Mouse Phenotype Consortium [72] have been associated with glycoenzymes and glycomotifs. This association connects GlycoMotif to the Mammalian Phenotype (MP) Ontology [73], and to the Human Phenotype Ontology [74] by association.

The majority of the resources use Disease Ontology (DO) [75] and Mondo Disease Ontology (Mondo) [76] for disease annotations, Uberon [77] for anatomy, BRENDA Tissue Ontology [78] for tissue, and Cellosaurus [79], Cell Line Ontology (CLO) [80], Cell Ontology (CL) [81], and Experimental Factor Ontology (EFO) [82] for cell lines.

#### Q4 conclusion

Participants agreed that it is necessary to harmonize or at the very least provide robust mapping tools for the standards used for glycan-related annotations while maintaining a mechanism to represent ambiguity in glycan structures. Although GlyTouCan provides a unique reference for glycan structures, it is not used by all resources as the primary glycan identifier. Many groups use ChEBI IDs instead. However, ChEBI relies on GlySpace group members and others to provide the GlyTouCan cross-references and improve ChEBI’s carbohydrate ontology. SNFG work has catalyzed the glycoinformatics community to use a common graphical representation of glycans. Close collaboration between NCBI, SNFG, and GlySpace Alliance is expected to further improve SNFG usage in publications. The GlycoMotif use case has established that glycan structures can be connected to phenotype ontologies, but these ontologies are still lacking terms relevant to glycosylation. Glycan-specific ontologies can provide a better understanding of glycan function within larger biological contexts. A vast majority of glycoinformatics resources provide Application Programming Interfaces and the option of using SPARQL queries (https://www.w3.org/TR/sparql11-query/). These options could be better utilized as common standards emerge from workshops such as the one described here.

### Other relevant resources

It is important to note there are several resources that were not represented at the workshop that contribute directly or indirectly to glycan function annotation. For instance, the PDB recently completed a Carbohydrate Remediation Project (https://www.wwpdb.org/documentation/carbohydrate-remediation), resulting in the development of a standard representation and validation framework for carbohydrate 3D structures within the PDB Core Archive [83, 84]. The Carbohydrate Structure Databases include data on manually curated natural carbohydrates from prokaryotes, plants, and fungi [85]. The O-GlcNAc Database provides an inventory of human O-GlcNAcylated proteins, their O-GlcNAc sites, identification methods, and corresponding references [34]. KEGG GLYCAN offers a collection of glycan structures, integrated with other KEGG resources, that allows for the examination of glycan-related pathways and networks [86]. The GlycoGene Database is a manually curated database of genes related to glycan synthesis [87]. Several other resources provide contextual information related to glycan function such as MetaboLights, a database for metabolomics studies and derived information [88], and Reactome, a manually curated and peer-reviewed pathway database [89]. A comprehensive list of glycoinformatics resources can be found in the Glycoinformatics chapter of Essentials of Glycobiology [90].

## Workshop conclusions and future directions

This inaugural workshop on annotating glycan functions significantly improved our understanding of data collection and curation strategies across resources and also highlighted current deficits in the organization of annotations related to glycans and glycoconjugate function. It became evident that a harmonized, international effort is needed for the comprehensive capture of information in this domain. Several collaborative initiatives were proposed, such as the co-curation of research papers by multiple groups and the establishment of dedicated communication channels to facilitate user queries on glycan function curation and expert responses to those queries. Various groups expressed interest in forging targeted curation efforts. For instance, the GO Group expressed its willingness to collaborate with glycan biology experts to ensure the accuracy and completeness of glycan-related content within GO. This community is committed to soliciting feedback that can make their functional annotations more beneficial to the broader scientific community.

To achieve a cultural shift in data collection practices, it is vital for researchers to actively document glycan information, experimental details, and sample metadata at the data generation stage, paving the way for effective curation. Additionally, there is a recognized need to foster awareness and provide training within the scientific community to underscore the value of curation, which may not currently be apparent to all. The tremendous potential of Large Language Models (LLMs) cannot be ignored. LLMs potentially could be used to retrieve and summarize data from publications and, in the future, help in standardization, such as finding all different names or representations of a given glycan. However, there is still an effort needed from different stakeholders (e.g. authors, publishers, resource/tool developers, and funding agencies) to better structure and standardize the glycan information in the literature to help drive these developments. Two related and potentially parallel strategies were discussed at the workshop. One strategy would have the author/researcher bring data to the resources which would provide tools to help with standardization for annotation. The other would enlist the community to help curate publications to establish glycan function-related annotations. An incentive for both approaches could be to give contributors acknowledgment through ORCID (https://orcid.org), allowing them to cite their contributions. Both models involve significant outreach and applying guidelines at strategic levels, ideally beginning before, during, or soon after publication. Thus, journal cooperation is also critical. Funding agencies should play a role in this effort as well, by supporting bioinformatic efforts that target increased data connectivity and, perhaps, by requesting that grant application reviewers evaluate whether applicants have followed community data standards in their recent publications.

The participants in this workshop were unanimous in their support for future opportunities to gather, evaluate progress, and propose strategies to collect and standardize annotations related to glycan function. They suggested that experts in glycan and glycoconjugate functions should meet next to propose useful annotation terms and concepts that can facilitate progress by informaticists. To that end, a second workshop on glycan function annotation titled “Defining Glycan Functions” was organized and held in conjunction with the 2023 Society for Glycobiology meeting in Hawaii (https://wiki.glygen.org/Glycan_Function_Workshop_at_SfG_2023). The second workshop enlisted experts in various domains of glycobiology to participate in discussions and contribute their insights toward the development of a robust framework comprising terms and concepts that link glycan functions with glycan structural features, motifs, and patterns. The discussion and outcomes of the second workshop, like this workshop, will be published for public comment and will serve as a framework for a future workshop that will bring together the glycoscience and bioinformatics participants for an event that promises to be a significant milestone in advancing the standardization of glycan function annotations and their representation across major biology and biomedical resources. We encourage the scientific community to provide input that can guide curation. The best way to provide feedback to improve and guide the glycan function curation efforts is to contact the GlySpace Alliance members via their contact page (http://www.glyspace.org/contact.php). Additionally, community members can also contact the Society for Glycobiology at SFG@glycobiology.org to reach out to a larger group of glycobiology researchers.

## Supplementary Material

baae073_Supp

## Data Availability

No new data were generated or analyzed in support of this research. The workshop agenda can be accessed at https://wiki.glygen.org/Glycan_Function_Workshop_2023.
