# Supplementary material for: Functional implications of glycans and their curation: insights from the workshop held at the 16th Annual International Biocuration Conference in Padua, Italy
Source: Database (Oxford). 2024 Aug 13;2024:baae073. doi: 10.1093/database/baae073 (PMC11321244; doi:10.1093/database/baae073)
Supplement: baae073_Supp [file baae073_supp.zip › suppl_data/SupplementaryFile1_final.docx]

| Supplementary file 1. Descriptions of resources and projects represented in the workshop. | |
| --- | --- |
| Resource/Project | Resource/Project Description |
| ChEBI | Chemical Entities of Biological Interest (ChEBI) (<https://www.ebi.ac.uk/chebi/>) is an open-access database and ontology of chemical entities. The chemical entities in question are either natural products or synthetic compounds used to intervene in the processes of living organisms. ChEBI is widely used as a small-molecule reference database by several other leading resources such as UniProt, GO, Rhea, and MetaboLights. To date, ChEBI contains over 60,700 fully annotated entries and a further 101,000 entries that are partially annotated. Each data item is fully traceable and explicitly referenced to the original source and all data in the database is freely accessible and downloadable without restriction. |
| Gene Ontology | The Gene Ontology (GO) knowledgebase (<http://geneontology.org>) is a comprehensive resource concerning the functions of genes and gene products (proteins and noncoding RNAs). The GO knowledgebase consists of three components: the Gene Ontology, an ontology of all known gene functions and the relationships between them; GO annotations, evidence-based assertions that associate a specific gene with a specific function from the ontology; and GO Causal Activity models (GO-CAMs), models of biological pathways/processes describing how different gene products function together in a biological system. |
| Glybrary | Glybrary (<https://glybrary.com/>) is a data management tool being developed to capture and curate experimental and sample metadata upstream at the time of performing experiments or obtaining samples. Samples or probes such as glycoconjugates, proteins, antibodies, chemicals, and biospecimens can be entered. Glybrary offers such entries to be linked to external databases in the field by the creator, thereby encouraging curation of the metadata around such samples. Glybrary is also developing an experiment cataloging system to capture experimental metadata and raw results files. |
| Glyco.me, GlycoDomainViewer | The Glyco.me and GlycoDomainViewer resources (<https://glyco.me>) are knowledgebases designed to help researchers navigate the glycome. The resources cover information primarily about the human glycome and glycoproteome, including information about the genes that encode for the glycosylation machinery, glycosylation pathways, and glycoproteins and glycosites on over 14,000 human proteins (at the GlycoDomainViewer resource). |
| Glyco@Expasy | Glyco@Expasy (<https://www.expasy.org/resources/glyco-expasy>) centralizes references to strictly web-based glycoinformatics resources, whether hosted or not on the SIB Expasy server. This portal aims to make glycobiology more accessible to scientists in both the glycosciences and the protein sciences. It advocates for presenting glycans as mediators of protein−protein interactions. The main in-house resources are GlyConnect, a knowledgebase of glycoproteins and glycans, and UniLectin, a knowledgebase of glycan-binding proteins with their ligands, cross-referencing each other with an ontology-based substructure search (GlycoQL). |
| GlycoEnzOnto | GlycoEnzOnto (<https://github.com/neel-lab/GlycoEnzOnto>) is an ontology describing the organization of 403 human glycoEnzymes that participate in all aspects of mammalian glycosylation. These enzymes are curated along 139 glycosylation pathways, 134 molecular functions, and 22 cellular compartments. The pathways described in the ontology regulate nucleotide-sugar metabolism, glycosyl-substrate/donor transport, glycan biosynthesis, and degradation. The ontology describes the role of each enzyme in the glycosylation initiation, elongation/branching, and capping/termination phases. IUPAC linear strings present systematic human/machine-readable descriptions of individual reaction steps and enable automated knowledge-based curation of biochemical networks. All GlycoEnzOnto knowledge is integrated with the Gene Ontology biological processes. GlycoEnzOnto enables improved transcript overrepresentation analyses and glycosylation pathway identification. The resource aims to present a holistic view of cellular glycosylation for systems-level analyses. While curated for humans, the basic knowledgebase may be extended to other species. |
| GlycoMotif | The GlycoMotif (<https://glycomotif.glyomics.org/>) glycan determinant and motif resource unifies a variety of glycan motif lists in one place and provides precomputed alignments of all motifs with GlyTouCan accessions. Additional motifs are used to help classify glycan structures into types and subtypes. Enzyme annotations on glycan structures and motif alignments to structures are used to associate glycoenzymes with motif residues, so gene-based functional annotations of glycoenzymes can be associated with functional glycomotifs. |
| Glycosciences.de | Glycosciences.de is a web portal featuring various databases and online tools for glycobiology. The focus is on 3D structural data, but other aspects are covered as well. One of the major sources of data is the Protein Data Bank (PDB). Tools for detection and validation of carbohydrate data in PDB entries (pdb2linucs and pdb-care, respectively) enable a mostly automatic parsing of the weekly released new entries in the PDB. Only those cases where potential issues are detected need to be reviewed manually. Glycan data from the scientific literature are also entered into the database. This process, however, needs manual curation. |
| GlycoShape | The GlycoShape platform (https://glycoshape.org/) provides users with a wealth of information of 3D structural information of glycans determined from molecular dynamics (MD) simulations. Extensive MD simulations of these free glycans are conducted to sample the flexibility and conformations of the glycan. The conformational ensembles from these multi-microsecond MD simulations are clustered into representative conformations, which are then available in a comprehensive open-access database, called the GlycoShape Glycan Database (GDB). The GDB contains structural data for over 435 glycans obtained from over 1 ms of cumulative MD sampling. The GlycoShape platform also includes a number of useful structural tools. For example, in GlycoShape structural data from the GDB can be used to rebuild glycoproteins to their functional, native state through a bespoke tool, named Re-Glyco. Additionally, Re-Glyco can be also used to predict occupancy of N-glycosylation sequons, using a tool named ‘GlcNAc Scanning’. |
| GlyCosmos | GlyCosmos (<https://glycosmos.org/>) is a Web portal for glycosciences, providing glycan-related omics data through the use of Semantic Web technologies. It includes four subsections: repositories (including GlyTouCan, GlycoPOST and UniCarb-DR), data resources, standards, and tools. Data resources are classified according to data type, such as glycogene, glycoprotein, glycolipid, disease, pathway, etc., and is the core part of GlyCosmos, collating data from the GlySpace Alliance as well as other data sources. Tools include glycan drawing tools (based on SNFG standards), GlycoMaple for visualization of glycan-related gene expression data, among others. |
| Glycowork | Glycowork (<https://github.com/BojarLab/glycowork>) is an open-source Python package containing code to analyze glycans at the structural level, draw glycans, interpret glycomics data, and more. Glycowork acts as a data store and is associated with a manually curated glycan database called Sugarbase. Glycan structures and their related biological sources are extracted from publicly available resources including databases and published papers, often manually transcribed from published images to text-based notation. |
| GlyGen | GlyGen (<https://glygen.org/>) is a data integration and dissemination project for carbohydrate and glycoconjugate-related data. GlyGen retrieves information from literature, and collaborates with individual laboratories and multiple international data sources, and integrates and harmonizes this data. This web portal allows exploring this data and performing unique searches that cannot be executed in any of the integrated databases alone. Key glycan function-relevant projects include the GlycoMotif collection, Glycan Dictionary, Sandbox, GlycoTree, and Glycan structure and function-related text mining. |
| GlyGen Sandbox | The GlyGen Sandbox (<https://sandbox.glyomics.org/>) for glycoenzymes maps human and mouse glycosylation enzymes to the residues of defined glycan structures, using a curated super-tree of potential glycan structures. |
| GlyTouCan | GlyTouCan (<https://glytoucan.org/>) is the international glycan repository, which can take as input any “glycan structure” and assign it a unique accession number. Glycan structures include monosaccharide compositions (as often identified by mass spectrometry, for example), glycan fragments, glycan topologies where monosaccharides and their glycosidic linkages are known, but the exact conformation of the linkages are unknown, and fully-defined glycans, where all chemical details of the structure have been identified. GlyTouCan depends on the WURCS representation of glycan structures to ensure that all structure is uniquely distinguished, allowing the assignment of GlyTouCan IDs. |
| GNOme | GNOme (<https://gnome.glyomics.org/>) is an OBOFoundry-based ontology for GlyTouCan identifiers organized by subsumption - the degree of structural specification. GlyGen uses the GNOme ontology for subsumption exploration and for propagating annotations (including species and glycan classifications) from more characterized structures to less characterized structures. |
| IMGT | IMGT® (<https://www.imgt.org/>) extends access to specialized databases and tools tailored to the study of the adaptive immune response. Adhering to the tenets of IMGT-ONTOLOGY, IMGT® ensures the provision of FAIR (Findable, Accessible, Interoperable, Reusable) data. Through close collaboration with INSDC, IMGT® establishes a unified gateway to genes and alleles of immunoglobulins (IG) and T cell receptors (TR). Furthermore, IMGT® delivers a wealth of information concerning the 3D structures of IG and TR. In partnership with SIB, IMGT has curated a catalog of glycosylated antibodies contained within its repository, aiming to standardize nomenclature based on established terminology. This collaborative effort has spurred a joint investigation focused on identifying shared PDB structures across both databases and forging connections between the IMGT/3Dstructure-DB and the GlyConnect database. |
| Immune Epitope Database and Analysis Project | The Immune Epitope Database (IEDB) (<https://www.iedb.org/>) is a freely available resource funded by NIAID. It catalogs experimental data on antibody and T cell epitopes studied in humans and other animal species in the context of infectious disease, allergy, autoimmunity, and transplantation. The IEDB adds new data weekly as new publications are added to PubMed. Currently, the IEDB has data on 1,595,239 peptidic and 3,185 non-peptidic epitopes, derived from 24,039 publications. The IEDB also hosts epitope prediction and analysis tools and has a companion site, CEDAR (funded by NCI), which houses cancer epitopes. |
| IntAct | The IntAct molecular interaction database (<https://www.ebi.ac.uk/intact>) is a curated resource of molecular interactions, derived from the scientific literature and from direct data depositions. The resource is mostly focused on protein-protein interactions but has decent coverage on nucleic acids and small molecules including glycans, and drugs. As of July 2023, IntAct provides more than one million binary interactions, curated by twelve global partners of the International Molecular Exchange Consortium (<https://www.imexconsortium.org/>) for which the IntAct database provides a shared curation and dissemination platform. |
| MatrixDB | MatrixDB (<http://matrixdb.univ-lyon1.fr/>) is an interaction database focused on the extracellular matrix (ECM). MatrixDB manually curates protein-protein and protein-glycosaminoglycan interactions involving at least one ECM constituent. MatrixDB has adopted the standards defined by the Molecular Interactions group of the HUPO Proteomics Standards Initiative (HUPO-PSI) for curating and exchanging interaction data. MatrixDB belongs to the International Molecular Exchange (IMEx) consortium (<https://www.imexconsortium.org/>) and follows its curation rules. MatrixDB offers users the possibility to generate tissue-specific or cell-specific interaction networks, thanks to the integration of gene expression data (https://www.ebi.ac.uk/gxa/home) and quantitative ECM proteomic data (http://matrisomeproject.mit.edu/ecm-atlas), and to export these networks for computational analysis. Users can also browse MatrixDB using a single criterion or combined criteria (e.g., interaction detection method, diseases, biological processes, molecular functions, or publications) to build criteria-specific interaction networks. |
| Privateer | The Privateer software (<https://github.com/glycojones/privateer>) provides methods to analyze, validate, and represent free and covalently attached glycans in macromolecular structures. It interacts with locally cached copies of both GlyToucan and GlyConnect via WURCS strings to provide experimental references to glycan structures modeled in 3D. It relies on the Cremer-Pople algorithm to calculate ring conformations, which are compared to a database of pre-calculated minimal-energy conformations to identify infrequent ring puckers, as these usually flag up interesting (i.e. a pyranoside in the -1 subsite of an enzyme) or wrong models (i.e. distorted in the absence of supporting electron density). Restraint dictionaries are generated whenever wrong models are detected: these helps refine the structures into their minimal energy pucker. *O-,* C- *and N*-glycans among others are represented using an annotated version of the SNFG, using orange backgrounds to highlight linkages or pyranosides in high-energy conformations. Privateer is free software, distributed by CCP4 and CCP-EM. |
| PubChem | PubChem (<https://pubchem.ncbi.nlm.nih.gov/>) is an open chemistry database at the National Institutes of Health (NIH). “Open” means that you can put your scientific data in PubChem and that others may use it. Since its launch in 2004, PubChem has become a key chemical information resource for scientists, students, and the general public. Each month PubChem website and programmatic services provide data to several million users worldwide. |
| Rhea | Rhea (<https://www.rhea-db.org/>) is an expert-curated knowledgebase of biochemical reactions that uses the chemical ontology ChEBI (Chemical Entities of Biological Interest) to represent reaction participants. Rhea covers enzymatic reactions and transport reactions, including but not limited to those described by the Enzyme Classification of the IUBMB, as well as reactions that occur spontaneously in biological systems. Rhea is now the reference vocabulary for enzyme annotation in the UniProt Knowledgebase (UniProtKB). |
